# Labeled entities from social media data related to avian influenza disease

**DOI:** 10.1016/j.dib.2022.108317

**Published:** 2022-05-27

**Authors:** Camille Schaeffer, Roberto Interdonato, Renaud Lancelot, Mathieu Roche, Maguelonne Teisseire

**Affiliations:** a INRAE, Montpellier F-34398, France; b CIRAD, Montpellier F-34398, France; c TETIS, Univ. Montpellier, AgroParisTech, CIRAD, CNRS, INRAE, Montpellier 34090, France; d ASTRE, Univ. Montpellier, CIRAD, INRAE, Montpellier 34398, France

**Keywords:** Text mining, Named entity recognition, Social networks, Epidemiology, Avian influenza

## Abstract

This dataset is composed by spatial (e.g. location) and thematic (e.g. diseases, symptoms, virus) entities concerning avian influenza in social media (textual) data in English. It was created from three corpora: the first one includes 10 transcriptions of YouTube videos and 70 tweets manually annotated. The second corpus is composed by the same textual data but automatically annotated with Named Entity Recognition (NER) tools. These two corpora have been built to evaluate NER tools and apply them to a bigger corpus. The third corpus is composed of 100 YouTube transcriptions automatically annotated with NER tools. The aim of the annotation task is to recognize spatial information such as the names of the cities and epidemiological information such as the names of the diseases. An annotation guideline is provided in order to ensure a unified annotation and to help the annotators. This dataset can be used to train or evaluate Natural Language Processing (NLP) approaches such as specialized entity recognition.

## Specification Table

**Table:** 

Subject	Data Science: Data Mining and Statistical Analysis
Specific subject area	Identify epidemiological events on social networks
Type of data	Table
How data were acquired	Data are the result of a manual and automatic annotation of spatial and thematic entities. The corpora 1 and 2, including 80 transcriptions and tweets, were automatically extracted from the platforms YouTube (December 2020-January 2021) and Twitter (March-April 2021). The corpus 3, including 100 transcriptions, was automatically extracted from the YouTube platform (February 2019 to October 2021). These three corpora were preprocessed after the extraction. Preprocessing programs are included in the dataset. Manual annotations were performed in accordance to the annotation guideline available within this dataset^1^. For right privacy, the content of tweets is not available in the dataset, instead there is the id of the tweet. On the other side, transcription texts are included.
Data format	Raw and Standardized
Parameters for data collection	The collection of texts respects two conditions: the texts have to be written in English and the term ‘avian influenza’ must be present in the text body for the tweets and in their title for the YouTube videos (i.e. primary data sources). The textual transcriptions collected from YouTube have been normalized by adding punctuation and capitalization, and by correcting transcription and lexical errors for specific terms (e.g. disease names). For Twitter data, non-Unicode characters (emoticons, etc.) have been removed, as well as correcting lexical errors of specific terms (i.e. secondary data sources).
Description of data collection	The dataset is constituted of 5 table files (i.e. 3 table files for YouTube transcriptions data and 2 table files for Twitter data). The table files describe each data through a set of features.
Data source location	The data are hosted on the INRAE Dataverse. The data were manually collected within the UMR TETIS, Univ. Montpellier, AgroParisTech, CIRAD, CNRS, INRAE, Montpellier, France in the context of the MOOD project^2^.
Data accessibility	Repository name: Data INRAE (Dataverse). Data identification number: 10.15454/GR5EFS. Direct URL to data: https://doi.org/10.15454/GR5EFS

## Value of the Data

• This dataset contributes to the available resources for Natural Language Processing (NLP) on specialized domains and more precisely in the field of epidemiology.
• This dataset is useful for computer scientists for NLP and data mining tasks.
• This dataset can be used for evaluation or training purposes for the entity recognition task.
• The annotators have identified a variety of entities (e.g. diseases, symptoms, virus, hosts). These entities are relevant to recognize epidemiological information.

## 1 Data Description

After an extraction process from YouTube and Twitter (i.e. primary data sources), the dataset is constituted of five table data files (.tab) normalized (i.e. secondary data sources) and annotated (i.e. final data sources). One annotation guide (.pdf) details the instruction to the annotators as well as the choices that were made while designing the study.

The python codes to reproduce the transformation of the documents for the annotation step is available on github.^3^

The five data files are distributed as follows:

### 1.1 Manual annotation of spatial and thematic entities - Corpus 1 (small)

• A table data file containing the YouTube transcription data, manually annotated, from corpus 1, named corpus 1Y (datafile_1Y_manual_annotation.tab);
• A table data file containing the Twitter data, manually annotated, from corpus 1, named corpus 1T (datafile_1T_manual_annotation.tab).

### 1.2 Automatic annotation of spatial and thematic entities - Corpus 2 (small)

• A table data file containing the YouTube transcription data, automatically annotated, from corpus 2, named corpus 2Y (datafile_2Y_automatic_annotation.tab);
• A table data file containing the Twitter data, automatically annotated, from corpus 2, named corpus 2Y (datafile_2T_automatic_annotation.tab).

### 1.3 Automatic annotation of spatial and thematic entities - Corpus 3 (big)

• A table data file containing the transcriptions YouTube data annotated automatically from corpus 3, named corpus 3Y (datafile_3Y_automatic_annotation.tab).

The files from the three corpora include data organized in tables. An example of the YouTube transcription data row is given in Table 1 and an example of the Twitter data row is reported in Table 2.

**Table 1:** Example of a row in the transcriptions YouTube data files.

Features	Values
**id**	P5uhnL0Fu0I
**publication_date**	2021-02-09T14:21:34Z
**raw_text**	breaking news coming in bird flu hits delhi zoo [...]
**normalized_text**	Breaking news coming in Bird Flu, hits Delhi, Zoo [...]
**spatial_entity**	(‘Delhi’, ‘GPE’), (‘bhopal’, ‘GPE’)
**thematic_entity**	(‘bird flu’, ‘Disease or Syndrome’),
	(‘flu’, ‘Disease or Syndrome’), (‘coronavirus’, ‘Virus’)
**Example of text with**	[...] My colleague is joining us with more details on the reports that are
**recognized entities**	just coming in seven positive cases of **bird flu** [...]
**(in bold)**	[...] the poultry markets of **Delhi** were reopened.

**Table 2:** Example of a row in the Twitter data files.

Features	Values
**id**	1377388750834040000
**publication_date**	1 April 2021
**spatial_entity**	(Europe’, ‘GPE’)(‘Russia’, ‘GPE’)(‘UK’, ‘GPE’)
**thematic_entity**	(‘Avian influenza’, ‘Disease or Syndrome’)(‘bird flu’, ‘Disease or Syndrome’)

The data are described through a set of features from YouTube transcription data of the three corpora:

- id: the id of the data on the social media;
- publication_date: the date of publication of the data on the social media;
- raw_text: the raw text (no transformation) of the data;
- normalized_text: the normalized text (after applying transformations) of the data;
- spatial_entity: A list of spatial entities annotated, with their labels (GPE, LOC, FAC);
- thematic_entity: A list of annotated thematic entities, with their labels (Disease or Syndrome, Virus, Sign or Symptom).

The data are described through a set of features from Twitter data of both corpora:

- id: the id of the data on the social media;
- publication_date: the date of publication of the data on the social media;
- spatial_entity: A list of annotated spatial entities, with its label (GPE, LOC, FAC);
- thematic_entity: A list of annotated thematic entities, with its label (Disease or Syndrome, Virus, Sign or Symptom).

The annotation guideline (annotation_guide_spatial_thematic_entities.pdf) presents the instructions to the annotators. These instructions and the choices made are summarized in the next section. The annotation framework defines several tags to annotate the texts.

Spatial concepts are annotated with three tags:

• *GPE* (Geopolitical entity) is used to annotate entities representing countries, cities, states etc;
• *LOC* (Non-GPE locations) is used to annotate entities representing mountain ranges, bodies of water, etc;
• *FAC* (Faculty) is used to annotate entities representing buildings, airports, highways, bridges, etc.

Thematic concepts are annotated with three tags:

• *Disease or Syndrome* is used to annotate entities representing a disease or a syndrome;
• *Virus* is used to annotate entities representing a virus;
• *Sign or Symptom* is used to annotate entities representing a sign or symptom.

Number and distribution of annotated information in the corpora are given in Tables 4 and 5.

**Table 3:** Distribution of the data in the dataset files Corpus 1 (i.e. manual annotation).

	Man. Ann. YTb^a^	Man. Ann. Twi^b^	all Man. Ann.^c^
Target	10 transcriptions	70 tweets	80 texts
**SPATIAL**	**97**	**37**	**134**
- GPE	86	35	121
- LOC	11	0	11
- FAC	0	2	2
**THEMATIC**	**120**	**118**	**238**
- Disease or Syndrome	35	87	122
- Virus	62	30	92
- Sign or Symptom	23	1	24
**TOTAL**	**217**	**155**	**372**

a Manual Annotation - Youtube.

b Manual Annotation - Twitter.

c Manual Annotation - Total.

**Table 4:** Distribution of the data in the dataset files Corpus 2 (i.e. automatic annotation).

	Auto. Ann. YTb^a^	Auto. Ann. Twi^b^	all Auto. Ann^c^
Target	10 transcriptions	70 tweets	80 texts
**SPATIAL**	**78**	**53**	**131**
- GPE	70	49	119
- LOC	5	3	8
- FAC	3	1	4
**THEMATIC**	**73**	**54**	**127**
- Disease or Syndrome	43	49	92
- Virus	20	5	25
- Sign or Symptom	10	0	10
**TOTAL**	**151**	**107**	**258**

a Automatic Annotation - Youtube.

b Automatic Annotation - Twitter.

c Automatic Annotation - Total.

**Table 5:** Distribution of the data in the dataset files Corpus 3 - larger corpus and only automatic annotation.

Target	Automatic Annotation - YouTube - 100 transcriptions - 605 567 words
**SPATIAL**	**1926**
- GPE	1535
- LOC	369
- FAC	22
**THEMATIC**	**3028**
- Disease or Syndrome	1407
- Virus	1429
- Sign or Symptom	192
**TOTAL**	**4954**

## 2 Experimental Design, Materials and Methods

### 2.1 Acquisition and pre-processing

The corpora were obtained automatically from the platforms YouTube and Twitter, thanks to their dedicated APIs. The texts from the web were stored in.txt files, with the aim to obtain distinct files to annotate.

The pre-processing consists of four main steps applied on the raw corpora, detailed below. First, in order to optimize the recognition of entities, the raw text of these files has been normalized with the automatic addition of punctuation by using the python library punctuator^4^ and with the automatic addition of capital letters, by using the POS tagging provided by the python library SpaCy^5^.

Then, the text has been cleaned by deleting non Unicode symbols and eventual noise originated by the transcription process. As a final normalization step of the raw text, a correction of specific terms (e.g. disease names) is applied by using regular expressions [1].

After the normalization task, the manual and automatic annotation of the spatial and thematic entities can be performed on the text.

### 2.2 Manual data labeling

In order to have a unified annotation of the spatial and thematic entities, a guideline was created. This annotation guide was written by a specialist in NLP. Three other persons (specialists in epidemiology) validated and adjusted these choices. By following this guide, one person (NLP specialist) annotated manually the spatial entities on the raw text data of 70 tweets and 10 transcriptions. In the same way, one person (data scientist specialized in epidemiology) manually annotated the thematic entities on the same data. This process results in the corpus 1 (1Y and 1T) and represents the ground truth data.

### 2.3 Automatic data labeling

In parallel, the spatial and thematic entities were automatically annotated on the same raw text data (70 tweets and 10 transcriptions) with NER tools. We applied SpaCy [2] on the data to recognize the spatial entities and SciSpaCy [3] combined with UMLS [4] in order to recognize the thematic entities. The result of this process is the corpus 2 (2T and 2Y).

Once these two annotations (manual and automatic) were performed, we evaluated the automatic recognition of the spatial and thematic entities on corpus 2, by comparing the annotation results of the corpus 1 and the corpus 2, and measured the evaluation with three metrics: Precision, Recall and F-Measure.

The evaluation is presented in Table 6. The Precision value of spatial and thematic entity recognition is quite good, with a result of 0.7 and 0.6 respectively. The Recall for automatic recognition of thematic entities is low (0.2), the tool does not recognize many thematic entities that are not included in the thesaurus associated with SciSpaCy.

**Table 6:** Evaluation of the automatic recognition of spatial and thematic entities on corpus 1

	Precision	Recall	F-Measure
**SPATIAL**	**0.7**	**0.6**	**0.6**
- GPE	0.7	0.6	0.6
- LOC	0.6	0.3	0.4
- FAC	0	0	0
**THEMATIC**	**0.6**	**0.2**	**0.3**
- Disease or Syndrome	0.5	0.3	0.4
- Virus	0.6	0.1	0.2
- Sign or Symptom	0.5	0.3	0.4

We then apply the processes of *Acquisition and pre-processing* and *Automatic data labeling* on a larger corpus (corpus 3) called 3Y (100 transcriptions). This enables to check the scalability of the proposed process.

All text related information such as copyright, texts, authors list or references were discarded.

The dataset is composed of the extracted annotations. The extraction program, available in the dataset, preserves the text and adds the textual entities and their annotated categories at the end of the text.

## Ethics Statement

For Twitter data, we keep the message identifiers and the content of message is not stored in accordance to Twitter’s terms of use. The content of YouTube data (i.e. transcriptions) is anonymized by removing the user names and the names of person in transcripts (using SpaCy for name recognition).

No conflict of interest exists in this submission. The authors declare that the work described in this paper is original and not under consideration for publication elsewhere, in whole or in part. Its publication is approved by all the authors listed.

## CRediT authorship contribution statement

**Camille Schaeffer:** Methodology, Software, Investigation, Resources, Data curation, Writing – original draft. **Roberto Interdonato:** Methodology, Writing – review & editing. **Renaud Lancelot:** Methodology, Writing – review & editing. **Mathieu Roche:** Methodology, Writing – review & editing. **Maguelonne Teisseire:** Methodology, Writing – review & editing.

## Declaration of Competing Interest

The authors declare that they have no financial or personal interests that could influence the work reported in this paper.

## Data Availability

Labeled Entities from Social Media Data Related to Avian Influenza Disease (Original data) (Dataverse).

## Footnotes

1 https://doi.org/10.15454/GR5EFS

2 https://mood-h2020.eu/

3 DOI:10.5281/zenodo.6373107 https://github.com/interdonatos/epidemiological_social_media_data_collection

4 https://pypi.org/project/punctuator/

5 https://spacy.io/
